# From newborn screening to genomic medicine: challenges and suggestions on how to incorporate genomic newborn screening in public health programs

**DOI:** 10.1515/medgen-2022-2113

**Published:** 2022-05-07

**Authors:** Nicola Dikow, Beate Ditzen, Stefan Kölker, Georg F. Hoffmann, Christian P. Schaaf

**Affiliations:** Institute of Human Genetics, Heidelberg University, Heidelberg, Germany; Institute of Medical Psychology, Center for Psychosocial Medicine, University Hospital Heidelberg, Heidelberg, Germany; University Hospital Heidelberg, Center for Pediatric and Adolescent Medicine, Clinic I, Heidelberg, Germany; Baylor College of Medicine, Houston, Texas, USA; Jan and Dan Duncan Neurological Research Institute, Texas Children’s Hospital, Houston, Texas, USA

**Keywords:** genomic newborn screening, genomic medicine, public health, return of results, ELSA (ethical, legal, and social aspects)

## Abstract

Newborn screening (NBS) programs are considered among the most effective and efficient measures of secondary prevention in medicine. In individuals with medical conditions, genomic sequencing has become available in routine healthcare, and results from exome or genome sequencing may help to guide treatment decisions. Genomic sequencing in healthy or asymptomatic newborns (gNBS) is feasible and reveals clinically relevant disorders that are not detectable by biochemical analyses alone. However, the implementation of genomic sequencing in population-based screening programs comes with technological, clinical, ethical, and psychological issues, as well as economic and legal topics. Here, we address and discuss the most important questions to be considered when implementing gNBS, such as “which categories of results should be reported” or “which is the best time to return results”. We also offer ideas on how to balance expected benefits against possible harms to children and their families.

## Introduction

Newborn screening (NBS) programs are considered among the most effective public health programs of the twentieth and twenty-first centuries [1], [2]. They aim at early detection of treatable conditions in newborns [3]. In the late 1960s, only a limited number of diseases were considered eligible; the conditions were selected according to the original Wilson and Jungner criteria [4]. With the evolution of diagnostic possibilities with multiplex platforms such as tandem mass spectrometry (MS/MS) and new therapeutic options, screening panels have expanded widely and in an unharmonized way [5]. In 2006, the American College of Medical Genetics (ACMG) was commissioned to develop guidelines for state NBS. The expert panel presented new evaluation criteria and identified and recommended 29 early-onset conditions requiring timely intervention for which screening should be generally mandated in national NBS programs as well as additional conditions to be added optionally [6]. The statement also addressed state-based standardization of the entire process of NBS, from the communication of results to the collection of data about newborns with positive screening results. Together with the original Wilson and Jungner criteria, the 2006 ACMG report served as a basis for and the Recommended Universal Newborn Screening Panel (RUSP). Today, rapid development of genomic sequencing makes sequencing of all or a large number of genes available for high-throughput screening, and again the upcoming implementation of new technology in NBS demands careful consideration and development of standardized strategies. Furthermore, future genomic NBS (gNBS) programs have to deal with a variety of legal, ethical, and societal challenges and require the development of reliable algorithms for early prediction of disease severity.

A comparable development, where the implementation of new diagnostic and therapeutic options goes hand in hand, has been known in the molecular genetic diagnostics setting. At the beginning, a limited number of genes could be analyzed with Sanger sequencing, and after a decade of using panel analyses, genomic sequencing has become available in clinical care. In pediatrics, for example, sequencing is used for critically ill newborns in order to rapidly guide treatment decisions [7], [8] and very recently, an evidence based ACMG guideline recommended exome or genome sequencing as a first- or second-tier test for pediatric patients with congenital anomalies, developmental delay, or intellectual disability [9].

With the introduction of genomic sequencing into routine healthcare, central questions arise concerning technical challenges and clinical, social, ethical, intellectual, and psychological issues for families and healthcare providers, as well as economic and legal topics: (1) which results should be reported (2) to whom in the family, (3) which is the best time to return results, and (4) how can they be communicated to allow for informed decision making processes for those affected? gNBS is feasible and reveals clinically relevant conditions in newborns [10], but in contrast to the diagnostic setting for symptomatic patients, most newborns are healthy or asymptomatic, which enhances many of these challenges. The question if gNBS should become part of national programs needs to be weighed up against the advantages and disadvantages and framework conditions must be defined – it is time to initiate the scientific and public discussion and to develop best practices for incorporating gNBS in clinical care. We provide an overview of the most pressing questions to be considered when implementing gNBS, and we offer ideas on how to balance expected benefits against possible harms to children and their families.

## NBS versus gNBS

Current NBS programs consist of a limited number of tests, with each test providing a result that is specific for that particular disease [11]. Tests are based on diagnostic biomarkers which indicate functionally relevant dysfunction of a circumscribed pathway. New analyses can be added to the panel as soon as they fulfill strict criteria concerning severity and treatability of the disease to be screened for, as well as sufficient sensitivity and specificity of the test in a high-throughput setting. The question will be which test to add and how to ensure that parents understand the implications of the results. In contrast to current NBS programs, gNBS analyzes a large number of genes at the same time, producing an overwhelming amount of data [12]. Sensitivity and specificity of genomic sequencing are unknown for the detection of pathogenic variants in the majority of analyzed genes. Genes may be clinically relevant, but treatment options are not yet available for the majority of detectable conditions. Even when focusing on clinically relevant genes, variants within these genes may be of unclear pathogenicity. Therefore, in gNBS, the complexity of data interpretation raises a number of questions about the relation of potential benefit to negative consequences for newborns and their families [11], as decisions must be made about which results should be returned to the families and which information should not [13]. Comparing both strategies, it becomes apparent that NBS will not be replaced by gNBS in the near future. In the context of inherited metabolic diseases, the population-based NBSeq project evaluated whole exome sequencing (WES) as a method for gNBS and found a sensitivity and specificity of 88 % and 98.4 %, compared to 99.0 % and 99.8 % for MS/MS, respectively [14]. The authors concluded that WES may not be suitable as first-tier analysis, but could be used as a secondary test reduce false positive results, provide more specific diagnosis, or add disorders to the screening panel that are not detectable in biochemical analyses alone. This is in line with results from the BabySeq Project [15], demonstrating clear complementarity of information detected with both approaches. Therefore, both strategies may be of best use in combination.

## Diagnostic testing vs screening

Over the course of the last decades, genetic testing strategies have evolved from PCR-based single-gene analyses to next-generation sequencing (NGS), where a large number of genes are analyzed at the same time, producing massive amounts of data to be interpreted. Genetic analyses in healthy individuals were previously limited to the context of predictive genetic testing, where a single pathogenic variant was known in the family and healthy relatives asked for their own genetic status for that gene in order to plan life decisions or medical prevention programs.

**Figure 1:**
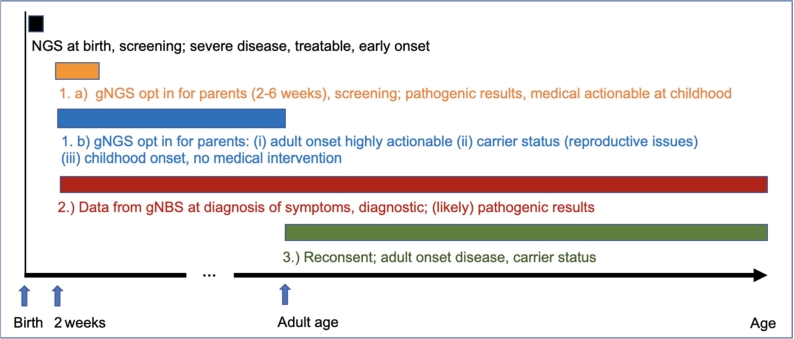
An approach to newborn screening and genomic medicine, considering different categories of results and the best time to return results to the families.

Genomic sequencing in a child with a medical condition has to be evaluated differently than genomic screening in a healthy or asymptomatic individual with an unknown but considerably lower *a priori* risk for a genetic disease than a clinically ill child [16]. In the first setting, if the child is already affected with a disease, the analysis may help to identify a molecular etiology of the symptoms and/or guide treatment options. Additional or incidental findings in genes not associated with the phenotype could be excluded from the report. Also, from a practical point of view, the clinical phenotype of the symptomatic child can be used to validate the genetic variants. In contrast, in the latter situation, where the child is asymptomatic, this clinical information is not available. It is not possible to focus the analysis on a specific group of genes that may be associated with a phenotype and the chances to validate variants of uncertain significance (VUS) are limited.

The return of genetic results from asymptomatic minors is particularly controversial. Most policy recommendations conclude that only information directly relevant for the child should be tested and reported to the parents. Focusing on a limited number of genes should reduce the burden of additional findings especially in children in order to respect nonmaleficence, beneficence, autonomy, and the right to an open future [13], [17], [18], [19], [20].

## A blueprint

Assuming that certain categories of data within the gNBS may be relevant to “avoid, treat, or prevent a genetic disease” (GenDG; Gendiagnostikgesetz) and that these findings could also be used to “guide a patient’s care through life” by interrogating the genomic data whenever clinically relevant [16], the most challenging issue will be defining what information should be returned to the parents or screened individuals at what point in life. The birth of a child is certainly an emotional moment for the parents and therefore parents may not have sufficient time to weigh up their decisions related to gNBS and informed consent. Not all information is immediately relevant to the child and there are national recommendations and laws regulating the analysis, consent, and return of genetic results in children. Schaaf et al. [21] have proposed an approach for the incorporation of genomic sequencing into NBS programs. They propose the continuation of conventional and well-established NBS at birth with focus on early-onset, severe treatable disorders, followed by optional gNBS at 2–6 weeks of age (Figure 1). The return of results for three categories of variants includes (1) immediately: pathogenic variants of medically treatable disorders in childhood; (2) with onset of symptoms: pathogenic or likely pathogenic variants for any genetic disease; and (3) at adult age and after re-consent: adult-onset pathogenic variants and information about carrier status.

The model represents a clear structure that resolves a number of the above-mentioned issues and benefits from a more refined view of the details taking into account the complexity of genetic results, the need for an informed consent, and healthcare and financial aspects, as well as issues related to legal aspects, data storage, and psychological consequences.

## Complexity of genetic results: What information should be returned?

It is obvious that the primary goal of gNBS should be to benefit the child. Therefore, the genes to be reported must be selected carefully.

1. By restricting reporting to clinically actionable childhood disorders, relevant information with high utility for the child may be missed. The notion of *utility* can be reduced to medical utility, but personal utility may be sufficient to justify screening for some individuals [22]. In the diagnostic context, the opinion that genetic results may be relevant even when there are no immediate treatment options is widely accepted. Considering medical utility, the boundaries between treatable and not treatable are not always easy to define; sometimes treatment or surveillance options are available for rare diseases, however their efficacy, especially long-term, has not yet been proven due to lack of data. Studies suggest that parents are interested in their newborn’s genetic variants, even if this information has no defined clinical utility [23]. The utility can also be extended to the family. The early and presymptomatic diagnosis in a child with Cohen syndrome, an autosomal recessive disorder with progressive blindness and intellectual disability, does not imply immediate treatment options for the newborn, but might be highly relevant for reproductive issues, as the chance of recurrence is 25 % for each future child of the respective couple. Parents may reproduce before clinical symptoms appear in the first affected child. As soon as the information is available in the gNBS data, not reporting the results could be an ethical issue. Therefore, parents should eventually get the right to receive this relevant data, if they request it. This is, however, associated with time-intensive counseling and re-analysis. Also, information about genetic predisposition to pharmacotoxicity should be available upon request. One could imagine a passport for each child in the medical record with drugs to be avoided; this seems more feasible than requesting a genetic report prior to every prescription of potentially harmful drugs.
2. *Carrier testing* for children is not recommended in most policy statements [24]. However, if carrier status is an incidental finding, the international statements are controversial [11]. The information might be relevant, again for reproductive questions of the parents. For example, the diagnosis of *X-linked* Duchenne muscular dystrophy might not be immediately relevant to a female newborn, and the return of results could be postponed into adulthood. If the mother of this newborn is a healthy carrier, a future son of hers is at risk of 50 % for this severe condition, which is lethal in adolescent age. If in autosomal recessive conditions only one variant is detected, a second variant in the second allele might have been missed due to incomplete sensitivity or a variant would not be reported as classified as VUS. These situations could be resolved by re-contact, re-phenotyping, assessment of family history, and/or genetic segregation analysis, which would enhance sensitivity and specificity of the analysis. In the BabySeq project, carrier status for *recessive* diseases were reported in 88 % of newborns [10]. Probably most healthcare systems will not be able to provide such an amount of effort. Then, a detailed section is needed in the informed consent on the technical and capacity limitations.
3. The more genetic analyses are performed, the more *VUS* are available in the data set. Most statements recommend not reporting VUS in the context of additional findings. A strategy of excluding VUS also from genomic screening results may therefore be necessary. In the BabySeq project, which includes healthy and ill newborns, Ceyhan-Birsoy et al. report VUS in the indication-based analysis only [10]. As in the diagnostic context, it would be helpful but time consuming to revisit the phenotype or add additional clinical evaluation as well as the family history, and eventually offer segregation analysis of the variant. Computational approaches in variant interpretation are rapidly evolving and in the near future, a genomic learning healthcare system may help to guide clinical decisions in the context of VUS in genes associated with early-onset treatable conditions in newborns [25].
4. Complexity is increased by *variability and incomplete penetrance* of variants in different genes, even within families. Penetrance is not always predictable, and even if estimates are available in a high-risk collective for variants in TP53 for example, the penetrance may differ in an asymptomatic unselected population such as healthy newborns. Clinical variability of rare diseases in the population may still be insufficiently known with distortion to severe “classical” manifestations. gNBS will need to be accompanied by longitudinal follow-up and registers following the FAIR data principles in order to assess associated penetrance and associated disease risks and to establish risk-adapted prevention programs for the selected diseases as well as reliable algorithms for early prediction of disease severity re-utilizing real-world patient data [26]. In addition to false positive results, there might be true positive results, e. g., a pathogenic variant in a disease-related gene, but with milder phenotype than anticipated. Polygenic risk scores have not yet been included in the context of gNBS but they will have to be considered in the analyses in order to predict lifetime risks.
5. There has been much discussion about variants in genes with *adult-onset disease*. If a pathogenic variant in *BRCA1* is known to run in the family, most guidelines state that presymptomatic testing should be deferred to adulthood. This is likely in the child’s best interest. The autonomy of the future young adult is protected and psychological, social, and legal aspects (e. g., insurability) are deferred. In contrast, in the context of gNBS, the family may be not aware of the variant in adult relatives [1]. Some authors argue for reporting adult-onset variants in order to protect relatives potentially at risk [27] or argue that the “benefit for the families” is more relevant than the single benefit for an individual [28]. As the information about conditions such as hereditary breast and ovarian cancer or Lynch syndrome is potentially life saving for a parent of the child, reporting this information is probably in the child’s best interest and not reporting in order to protect the child’s autonomy appears as ethically problematic. Ceyhan et al. [29] reported that approximately half of the parents decided to obtain data of adult-onset genes; in 3.5 % a pathogenic variant was detected, and in all three cases, one parent was affected and received recommendation for medical prevention programs.

While reporting of VUS and carrier status could be postponed, there is an urgent need to discuss the conditions to be included in gNBS with clinicians, researchers, ethicists, public health professionals, policy makers, and patients or patient representatives [5].

The gene list could be initially small and some highly relevant genes could simply be added to the current NBS programs, along with a published selection of genes from the ACMG list that are actionable in childhood and for which interventions are supported by evidence of at least moderate quality [30]. Alternatively, the list could be more extensive, including several hundred genes, as proposed by Ceyhan et al. [29].

The most feasible version for reporting variants during the first weeks of life in asymptomatic children might be a relatively small virtual panel containing actionable childhood-onset disease genes. Additional parental opt-in for return of results will have to be discussed, e. g., in three different categories as proposed by Milko et al. [31]: (1) adult-onset and highly actionable; (2) childhood-onset but no medical intervention available; and (3) carrier status with relevance for reproduction.

## Informed consent

NBS is seen in the best interest of the child and therefore widely applied without informed consent [5], and many states do not offer parents to opt out. In contrast, there is a broad consensus that informed consent is required for genomic analyses. Therefore, the two-step approach [21] could be helpful for practical implementation.

Based on such an approach after NBS, parents would be offered to opt in for gNBS. All parents who seek genetic information for their child would be informed about benefits and limitations of gNBS. Informed consent could be supported by online digital decision aids, as proposed and evaluated in the NC Nexus project for exome sequencing in newborns [31]. The time for the information about gNBS is not necessarily limited to the time of a child’s birth; teaching may begin as part of a population-based educational effort in schools, with the family doctors, or during pregnancy.

Technical limitations that should be mentioned include incomplete sensitivity and specificity of gNBS. The relatively common Fragile-X syndrome might be not detected due to the mutational mechanism, genomic deletions or duplications may be missed in exome-based approaches, the coverage may not be complete for all potentially relevant genes, and VUS could be re-evaluated as pathogenic variants in reanalysis based on new published data. Genes that are not evaluated as actionable and therefore not reported at the time of diagnosis may be later added to the list of relevant genes. A negative result does not exclude genetic disease. Consequently, information on sensitivity and specificity would need to be included into the consent documents and the resulting risk/benefit ratio should be communicated to the parents prior to consenting. Above this, informed consent should also include information on data storage, access, and protection (see below).

## Public health considerations

Implementation of such far-reaching diagnostic adaptations requires counseling and education of families and healthcare providers. In the BabySeq project, NGS revealed 9.4 % of newborns at risk of childhood-onset disease [10]. Disclosure of such information must be followed by additional medical treatments and/or surveillance programs. Only a limited number of experts is available to interpret complex data and communicate them to the families [32]. Also, data should be obtained using comparable pipelines, filter criteria, and annotation so that data are comparable between different individuals. This type of data harmonization needs to be initiated from the outset.

Not every healthcare system will be able to provide this service for all children or families. While it seems tempting in the context of genomic medicine to store genomic data in an individual’s health record to guide patients’ health decisions, we do not yet know if one single sequencing per person will be sufficient for the entire lifetime. Funding will be needed to store data and re-analyze the data; re-sequencing might be necessary as new and better sequencing technologies are developed.

When calculating the costs of implementation of gNBS, as in NBS, one should also evaluate and take into account how many lives can be saved or quality life years gained (including for family members after predictive testing), how many treatments or prevention programs can be offered, and how the knowledge of genetic information affects quality of life, both positively and negatively.

## Data storage and juridical issues

One sequencing per person may be sufficient for the entire lifetime or at least for many years. Data could be interrogated at different times in life. This requires DNA and data storage under special protection, as well as legal regulations on who may access data [33] in order to avoid legal disadvantages, especially in insurance and employment. This is relevant, as even pseudonymized genetic information could be used to track an individual and his or her family members.

Concepts ensuring privacy and confidentiality in studies should be considered when research questions arise over time and where the link to personal and family history might be relevant.

In Germany, the GenDG permits several types of insurance companies to request and use genetic information if the sum insured surpasses 300,000 euros or an annual annuity of 30,000 euros. A revision of the law seems worth consideration, especially if an individual has received genetic results at his or her own gNBS, the consent was signed by his or her parents, and he or she would suffer disadvantages for a test result that he or she never consented to.

A revision of the GenDG may also be necessary when adult-onset diseases should be reported in order to protect parents or other adult relatives of the child. In the current version of the law, analysis in the child is only applicable if it is relevant to the child itself or to family planning of his/her relatives. The admissibility of genetic analysis in minors might be expanded to adult-onset disease that is directly relevant to relatives.

Interestingly, these issues could be resolved after one generation, when all new parents already know their genomic profile from their own gNBS, if they want to know.

## Psychological aspects

Newborns have a right to privacy, a right not to know, a right to an open future. Potential harms discussed in the context of genetic analyses include a risk of anxiety, depression, and limited self-esteem, especially in children and in their parents. So far, little is known about if these harms really occur [34]. A recent study gave “no evidence that returning newborn genomic sequencing information has an unfavorable psychosocial effect on families” [35] and some studies report “no serious adverse psychological impacts from genetic testing and screening in children” [36]. Initial research suggests that the parents’ (mostly the mother’s) perception of risk, anxiety, and depression as well as behavior after a gNBS for single predefined diseases depends, among other things, on the parents’ prenatal mental health, on their education, social background, and living situation, and on the age of the child at the time of the screening [37], [38], [39], [40]. So far, no systematic data are available on parents or on how future family planning is affected through gNBS. Above this, it is unclear how parents estimate and comprehend the risk that their child will actually develop a disease. Individual risk perception depends on momentary influences such as stress levels, more stable predictors such as education and social background, and individual disease trajectories. Repetitive genetic counseling may be needed in order to minimize uncertainty and thus psychological distress. Studies focusing on long-term psychological consequences of gNBS will be needed. Shifting from an individual point of view to a more global societal perspective, one could imagine a dystopic development where the value of individuals (employment sector, insurances, self-esteem) could be influenced by a genetic risk profile or a shift towards a society that newly organizes itself in permanent response to risk, as discussed in “Risikogesellschaft” by Ulrich Beck in 1986. On the other hand, new minorities could emerge, with groups interested in every possible prediction and prevention and other groups who do not want to receive information. Society will have to deal with these discrepancies and recognize the right not to know also in the gNBS context.

## Conclusions

The recently developed concept of gNBS is methodologically feasible and provides highly relevant information for newborns and their families. Incorporating gNBS in the existing NBS approach may appear a logical next step for public healthcare; however, besides financial aspects, challenges, and the burden on healthcare providers, central questions on the ethical, psychological, and legal aspects of data storage, harmonization, access, and protection have to be addressed prior to implementation. A two-step approach that timely separates traditional NBS from gNBS has been suggested. Further, the amount of genomic information reported to the parents of healthy newborns could be limited to a carefully selected number of genes and variants that are expected to be medically actionable during childhood. At a later point, opt-in options for different categories of information relevant for the families could be provided upon request.

As the implementation of gNBS and genomic medicine bears substantial implications for societal perception of health risk and values, which go beyond the individual’s or family’s healthcare, a broad discussion in the general public is needed.
